# Safety and Immunogenicity of the Live Attenuated Varicella Vaccine in Vietnamese Children Aged 12 Months to 12 Years: An Open-Label, Single-Arm Bridging Study

**DOI:** 10.3390/v16060841

**Published:** 2024-05-24

**Authors:** Pham Van Hung, Le Thi Huong Giang, Phung Lam Toi, Vu Thi Minh Thuc, Bui Dang The Anh, Dinh Cong Pho, Pham Ngoc Hung

**Affiliations:** 1 The Company for Vaccine and Biological Production No. 1 (VABIOTECH), Ministry of Health, Hanoi 100000, Vietnam; hungpv@vabiotech.com.vn (P.V.H.); lehuonggiang.dk@vabiotech.com.vn (L.T.H.G.); 2 Health Strategy and Policy Institute, Ministry of Health, Hanoi 100000, Vietnam; toiphunglam@gmail.com; 3 Tam Anh General Hospital, Hanoi 100000, Vietnam; vuminhthuc2010@gmail.com; 4 Department of Epidemiology, Vietnam Military Medical University, Hanoi 100000, Vietnam; bdtanh@vmmu.edu.vn; 5 Department of Military Science, Vietnam Military Medical University, Hanoi 100000, Vietnam; dinhcongpho@vmmu.edu.vn; 6 Department of Education and Training, Vietnam Military Medical University, Hanoi 100000, Vietnam

**Keywords:** SKYVaricella vaccine, pediatric vaccination, varicella-zoster virus, immunogenicity, vaccine safety, Vietnam

## Abstract

Objective: This study aims to evaluate the safety and immunogenicity of the SKYVaricella vaccine in healthy Vietnamese children aged 12 months to 12 years. Methods: This open-label, single-arm study involved 201 children divided into two groups: 60 children aged 12 months to 5 years and 141 children aged 6 to 12 years. Safety was assessed through immediate reactions, solicited adverse events within 7 days, and unsolicited events up to Day 42. Immunogenicity was evaluated by seroconversion rates (SCR) and geometric mean titer (GMT) increments using fluorescent antibody-to-membrane antigen (FAMA) on the day of vaccination (D0) and 42 days after vaccination (D42). Results: All participants completed the follow-up. Immediate adverse events included pain (8.0%), redness (8.0%), and swelling (20.9%) at the injection site. Within 7 days, pain (17.9%) and swelling (12.4%) were mild and self-resolving. Unsolicited adverse events were infrequent and mild. Both age groups achieved 100% SCR. GMT of varicella-zoster virus antibodies increased from 1.37 (SD 1.97) at D0 to 18.02 (SD 2.22) at D42, a 13.12-fold rise. No Grade 3 adverse events were observed. Conclusion: The SKYVaricella vaccine shows a robust immunogenic response and favorable safety profile in Vietnamese children aged 12 months to 12 years. These findings endorse its potential inclusion in pediatric vaccination programs as a reliable preventive option against varicella.

## 1. Introduction

Varicella, commonly known as chickenpox, is a highly contagious disease caused by the varicella-zoster virus (VZV) [1]. It primarily affects children and is characterized by a fever and a distinctive rash of blisters. Although usually mild, chickenpox can lead to serious complications such as bacterial infections, pneumonia, and encephalitis [2]. Furthermore, it poses greater risks for individuals with weakened immune systems, pregnant women, and newborns. The global incidence of chickenpox is estimated at 140 million cases annually, resulting in approximately 4200 related deaths [3].

The integration of varicella vaccination into national immunization programs has significantly reduced both the occurrence and severity of the disease [4,5]. A live, attenuated varicella vaccine was developed in Japan in the 1970s and is effective in preventing primary varicella infection and possibly herpes zoster, a reactivation of VZV [6,7]. Despite the successes in disease prevention, global vaccine access disparities persist, highlighting the need for more widely available and affordable vaccination options.

The SKYVaricella vaccine is a live attenuated varicella vaccine that has been developed by SK Bioscience Co., Ltd. (Seongnam 13494, Republic of Korea). It was approved by the Ministry of Food and Drug Safety (MFDS) of Korea in 2018 and acquired pre-qualification certification from the WHO in 2019. The vaccine has shown promising results in children aged 12 months to 12 years, with high seroconversion rates and a well-tolerated safety profile [8]. However, it is important to note that the immunogenicity and safety of vaccines can vary across different ethnic and demographic groups, hence the need for region-specific studies [8].

In Vietnam, the peak incidence of varicella occurs during the hot and humid months from February to June, which makes virus transmission easier [9]. In 2018, over 30,000 cases were reported [10], highlighting the urgent need for effective vaccination strategies that are tailored to Vietnam’s specific circumstances. Given the approval of the SKYVaricella vaccine in Korea, the Vietnam ministry of health requires a bridging study to support the use of a vaccine in Vietnamese populations, in addition to the initial clinical trials in Korea.

This study aims to evaluate the safety and immunogenicity of the SKYVaricella vaccine in healthy Vietnamese children aged 12 months to 12 years by conducting an open-label, single-arm bridging study. The study’s findings will help to determine if SKYVaricella should be included in Vietnam’s National Expanded Program on Immunization. This could have a significant impact on public health in the region.

## 2. Materials and Methods

### 2.1. Study Design

This open-label, single-arm bridging study was designed to evaluate the immunogenicity and safety of the SKYVaricella vaccine in Vietnamese children aged 12 months to 12 years. Participants were continuously recruited into the study until the expected sample size was reached.

### 2.2. Participants

Eligible Vietnamese children aged 12 months to 12 years were enrolled and classified into two age groups: 12 months to 5 years and 6 years to 12 years. The participants were included in the study if they met all the following criteria: (1) healthy children whose parents or legal guardians consented to participate and were allowed to be monitored for the duration of the study; (2) guardian(s) of the participants and/or participants themselves should be capable of understanding the written consent form, and such form should be signed before the volunteer being included into this study; (3) after menarche, females who were confirmed to have a negative pregnancy test on the day of vaccination and agreed to apply birth control for 3 months after the vaccination.

Exclusion criteria included prior history of vaccination against varicella, history of chickenpox, an immunosuppressive condition, immunodeficiency, acute illness (including acute or chronic cardiovascular disorders, respiratory, endocrine, and neurological), or hypersensitivity to any vaccine component. The details of inclusion and exclusion criteria were described previously (NCT04384016). Next, only 35 study subjects were invited to participate in the immunogenicity assessment. The volume of each blood sample taken at the time of evaluation (at vaccination day [D0] and 42 days after vaccination [D42]) was 3 mL.

### 2.3. Vaccination Process

The SKYVaricella vaccine is derived from the already attenuated Oka strain of VZV manufactured by SK Bioscience. The original Oka strain (pOka) was isolated by Dr. Michiaki Takahashi in Japan in the early 1970s and then attenuated to create the Oka strain vaccine (vOka). This vOka strain was further developed and used by various manufacturers worldwide to produce varicella vaccines. In the development of the SKYVaricella vaccine, scientists in Korea used this established vOka strain as the foundation. They did not genetically modify the strain but relied on its proven efficacy and safety profile. The genetic analysis of the Oka/SK strain confirmed its close similarity to other commercial vaccine strains such as Oka/Merck (used in Varivax and Zostavax by MSD) and Oka/GSK (used in Varilrix by GSK), while being significantly different from wild-type strains like pOka.

The dose (0.5 mL) and single-dose regimen in this study were investigated according to the product dosing regimen approved by the Ministry of Food and Drug Safety (MFDS) of Korea. The dose and regimen of vaccination in this study have also been evaluated and approved by the Ministry of Health in Vietnam before implementation. Vaccination was performed under the supervision of trained healthcare professionals in a controlled clinical setting.

### 2.4. Follow-Up and Data Collection

Post-vaccination, participants were observed for immediate adverse events for 30 min. Parents or legal guardians were provided with diaries to record solicited adverse events for the following seven days. Unsolicited adverse events were monitored for up to six weeks post-vaccination. Follow-up visits were scheduled on Day 7 (early safety assessments) and Day 42 (safety and immunogenicity assessments).

### 2.5. Immunogenicity Assessment

The primary immunogenicity endpoint was the proportion of participants who achieved seroconversion at Day 42, as measured by the fluorescent antibody-to-membrane antigen (FAMA) test [11]. Seroconversion was defined as an increase in VZV antibody titers from <1:4 pre-vaccination to ≥1:4 post-vaccination. Secondary endpoints included geometric mean titers (GMT) of VZV antibodies at D0 and D42.

The varicella-zoster virus (VZV) expresses glycoproteins that act as antigens on the cell membrane. These glycoproteins combine with antibodies produced after varicella zoster vaccine inoculation. The FAMA assay uses this principle to detect viruses [11]. It involves inoculating a sensitive MRC-5 cell with a virus sample and using a fluorescein-labeled antibody to identify the virus antigen. The FAMA assay is considered the ‘gold standard’ for measuring VZV antibody levels and can be used as a reference for measuring other antibodies [12]. The presence of the virus and the specificity of the live varicella vaccine can be determined through this method.

### 2.6. Safety Assessment

Safety was evaluated by recording the incidence, type, and severity of adverse events following vaccination. Immediate reactions were documented, along with solicited adverse events within 7 days, and unsolicited events up to D42.

### 2.7. Statistical Analysis

Descriptive statistics were utilized for demographic information, seroconversion rates, and adverse events. The geometric mean titers (GMT) are presented along with the standard deviation (SD) and 95% confidence intervals (CI). The statistical significance of the increase in GMT from baseline was determined using a paired t-test, with a *p*-value of <0.05 considered significant. Analyses were conducted using SAS^®^ software, version 9.4.

### 2.8. Ethical Considerations

The study adhered to a protocol that was approved by the institutional review board and conducted in accordance with the Declaration of Helsinki, Good Clinical Practice guidelines, and local regulatory requirements. Written informed consent was secured from all participants’ guardians, and assent was obtained from older children where appropriate. The study protocol was approved by the IRB of the National Ethics Council (code: No. 28/CN-HĐĐĐ on 14 April 2020).

## 3. Results

### 3.1. Study Participants

**Figure 1:**
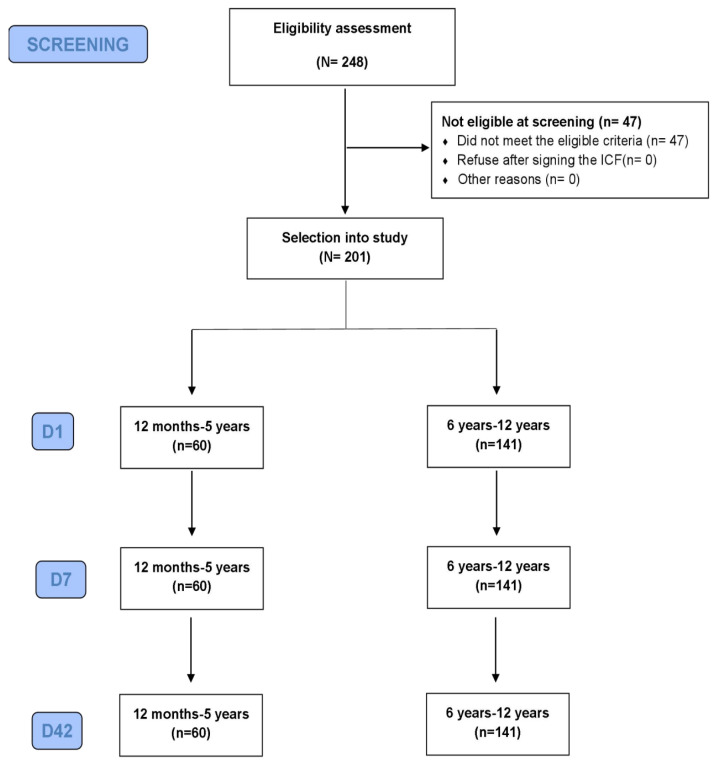
Study flow chart.

The study’s recruitment and enrollment process were conducted in Ha Nam province, following rigorous ethical standards, and ensuring informed consent. A total of 248 potential participants were screened, leading to the enrollment of 201 children who met the eligibility criteria. This cohort was stratified into two age groups: 60 children aged 12 months to 5 years and 141 children aged 6 years to 12 years, aligning closely with the study’s target enrollment of 200 participants. Throughout the 42-day post-vaccination observation period, all participants remained in the study (Figure 1). No deviations in the protocol were reported that indicate the consistent and controlled study execution throughout the entire period.

The demographic showed a balanced gender distribution (51.7% male, 48.3% female) and varied age groups. Significant differences regarding the mean height and weight were observed between the two age groups (Table 1).

### 3.2. Safety

The safety profile of the SKYVaricella vaccine was assessed through the examination of adverse events post-vaccination. This evaluation included immediate reactions within 30 min of vaccination and solicited adverse events reported within 7 days.

#### 3.2.1. Immediate Adverse Events

Table 2 presents the incidence and severity of these reactions. The results show that pain at the injection site was found in 3.3% of the 1–5 year-of-age group and 9.9% of the 6–12 year-of-age group, characterized solely as mild (grade 1). Redness at the injection site was more common among younger children, with a statistically significant difference noted between age groups (*p* < 0.05), yet primarily mild. Swelling was observed in 20.9% overall, marking the most prevalent local reaction, but remained mostly mild to moderate. The body temperature was slightly lower in the 1–5 year-of-age group (Table 2).

The comparison between the screening time (D0) and the end of the study (D42) regarding the temperature and pulse of participants is presented in Table 3. A slight, yet statistically significant, increase in mean body temperature to 36.8 °C from the screening time of 36.4 °C occurred. The mean pulse was also increased at the end of the study (D42) (Table 3).

#### 3.2.2. Solicited Adverse Events

Table 4 captures a range of solicited adverse events within 7 days post-vaccination, none of which exhibited statistically significant differences between the age groups (*p* > 0.05). Pain and swelling at the injection site were the most frequently reported mild adverse events. The rate of pain at the injection site and swelling among all participants were 17.9% and 12.4%, respectively. Fever was reported in 1.5% of all participants.

#### 3.2.3. Unsolicited Adverse Events

The study recorded 4 cases of mild adverse events (1.99%) and 4 cases of moderate adverse events (1.99%). There was an association between the presence of any adverse events and age groups (*p* = 0.01) (Table 5).

### 3.3. Immunogenicity

#### 3.3.1. Seroconversion Rates Post-Vaccination

Table 6 outlines the seroconversion rates observed within the study population. A 100% seroconversion rate was achieved across both cohorts by day 42 post-vaccination.

#### 3.3.2. Antibody Titers Enhancement

The geometric mean titer [GMT (SD)], which was 1.37 (1.97) at the D0, increased to 18.02 (2.22) on D42. The GMT raise at 42 days after vaccination (D42) compared to the baseline was 13.12-fold (2.02) (Table 7).

## 4. Discussion

This is a bridging study to provide safety and immunogenicity data for the SKYVaricella vaccine in 12-month-old to 12-year-old children in Vietnam. The study revealed no significant safety concerns for the SKYVaricella vaccine. Furthermore, the absolute rate of seroconversion measured via FAMA in the study (100%) shows that the vaccine produced the expected immunity.

The study results have provided promising findings that align with and expand upon the pivotal work conducted in Korea by Choi, et al. [8]. This direct comparison validates our findings and offers a comprehensive view of the vaccine’s performance across diverse demographic and epidemiological settings. Notably, the uniform 100% seroconversion rate observed in our study mirrors the high immunogenicity rates reported by Choi et al. [8]. This consistency highlights the SKYVaricella vaccine’s immunogenicity, which is vital for effective varicella infection control. Furthermore, the significant increase in GMTs emphasizes the vaccine’s ability to provoke a substantial antibody response, consistent with global research findings [13,14].

Our analysis of the safety profile showed predominantly mild adverse events, akin to those observed in the Korean study. This similarity underscores the vaccine’s appropriateness for pediatric use, reflecting a tolerable safety profile essential for broad acceptance and integration into national vaccination schedules.

Incorporating the SKYVaricella vaccine into national immunization schedules could significantly alleviate the varicella burden [15,16]. Our results, coupled with findings from Choi et al. [8] and supported by studies emphasizing the public health benefits of varicella vaccination [17,18], suggest the vaccine’s potential in comprehensive control strategies. This approach could lead to herd immunity and a decrease in disease transmission, marking a substantial advancement in public health efforts.

The strength of this study lies in its focused examination of the SKYVaricella vaccine’s safety and immunogenicity within a Vietnamese pediatric population. The study addresses a critical knowledge gap by providing essential data on the vaccine’s immunogenicity and safety within this specific population. This localized insight, supported by the World Health Organization’s call for region-specific evaluations [3], is a significant strength, ensuring the vaccine’s effectiveness across various settings. The methodological rigor, characterized by the thorough monitoring of adverse events and a comprehensive follow-up regime, ensures the reliability and applicability of the findings, enhancing the global vaccine safety literature [19]. Particularly notable is the 100% seroconversion rate observed, echoing the results of seminal studies and underscoring the SKYVaricella vaccine’s immunogenicity across different populations [8]. Furthermore, the study’s execution demonstrated exceptional participant retention and protocol adherence, reflecting its high quality and the validity of the data collected. These factors combined underscore the study’s vital contribution to expanding the evidence base supporting the SKYVaricella vaccine’s use in public health strategies for varicella prevention.

It is crucial to acknowledge the limitations of the study, such as the short-term follow-up and the absence of a control group. Future studies should explore long-term immunity and the vaccine’s efficacy against different VZV strains. Moreover, despite the proportion of participants between two age groups in the serology investigation being similar to that of the total sample size of the safety analysis, the small sample size is a limitation. Future studies might need to perform serology analysis for all participants. Also, future studies in diverse populations would further delineate the SKYVaricella vaccine’s universal efficacy and safety [14,20].

## 5. Conclusions

Our study demonstrates that the SKYVaricella vaccine is immunogenic and has a favorable safety profile in Vietnamese children. These findings indicate that the vaccine should be considered for inclusion in varicella vaccination programs in Vietnam to contribute to varicella prevention and control efforts. However, further long-term follow-up studies are necessary to comprehensively assess the vaccine’s safety and effectiveness in real-world settings.

## Figures and Tables

**Table 1:** The baseline demographic characteristic of participants.

Characteristics	1–5 Years Old (n = 60)	6–12 Years Old (n = 141)	Total (n = 201)
**Sex, n%**			
Male	34 (56.7%)	70 (49.6%)	104 (51.7%)
Female	26 (43.3%)	71 (50.4%)	97 (48.3%)
**Age, years**			
Mean (SD)	3.6 (1.4)	9.4 (2.1)	7.7 (3.3)
Median (min–max)	3.5 (1–5)	9.0 (6–12)	8.0 (1–12)
**Weight, kg**			
Mean (SD)	16.0 (4.1)	31.0 (9.4)	26.5 (10.7)
Median (min–max)	15.8 (10–29)	29.8 (14–57)	24.2 (10–57)
**Height, cm**			
Mean (SD)	99.5 (13.5)	134.6 (13.1)	124.1 (20.8)
Median (min–max)	103.5 (73–122)	134.0 (104–164)	126.0 (73–164)
**BMI, kg/m^2^**			
Mean (SD)	16.1 (2.0)	16.7 (2.8)	16.5 (2.6)
Median (min–max)	15.9 (13–24)	16.2 (11–24)	16.1 (11–24)
**Temperature, °C**			
Mean (SD)	36.4 (0.2)	36.4 (0.2)	36.4 (0.2)
Median (min–max)	36.4 (36–37)	36.4 (36–37)	36.4 (36–37)
**Pulse, beats/min**			
Mean (SD)	94.1 (17.0)	85.5 (17.3)	88.1 (17.6)
Median (min–max)	97.0 (56–136)	84.0 (51–129)	87.0 (51–136)
**Enrolled into Immunogenicity study, n%**			
No	50 (83.3%)	116 (82.3%)	166 (100%)
Yes	10 (16.7%)	25 (17.7%)	35 (17.4%)

**Table 2:** Incidence and severity of immediate adverse events post-vaccination by age groups.

Adverse Events	1–5 Years Old (n = 60)	6–12 Years Old (n = 141)	Total (n = 201)
**Pain at injection site**	2 (3.3%)	14 (9.9%)	16 (8.0%)
Grade 0	58 (96.7%)	127 (90.1%)	185 (92.0%)
Grade 1	2 (3.3%)	14 (9.9%)	16 (8.0%)
Redness (*)	9 (15.0%)	7 (5.0%)	16 (8.0%)
Grade 0	51 (85.0%)	134 (95.0%)	185 (92.0%)
Grade 1	2 (3.3%)	5 (3.5%)	7 (3.5%)
Grade 2	5 (8.3%)	1 (0.7%)	6 (3.0%)
Grade 3	2 (3.3%)	1 (0.7%)	3 (1.5%)
**Redness diameter in cm**			
Mean (SD)	19.1 (7.5)	14.6 (9.9)	17.1 (8.6)
Median (min–max)	20.0 (10–30)	10.0 (7–35)	15.0 (7–35)
**Swelling**	14 (23.3%)	28 (19.9%)	42 (20.9%)
Grade 0	46 (76.7%)	113 (80.1%)	159 (79.1%)
Grade 1	8 (13.3%)	8 (5.7%)	16 (8.0%)
Grade 2	3 (5.0%)	18 (12.8%)	21 (10.4%)
Grade 3	3 (5.0%)	2 (1.4%)	5 (2.5%)
**Swelling diameter in cm**			
Mean (SD)	14.9 (8.6)	17.0 (7.3)	16.3 (7.7)
Median (min–max)	11.0 (5–30)	17.5 (8–35)	15.0 (5–35)
**Fever**	0 (0%)	2 (1.4%)	2 (1.0%)
Grade 0	60 (100%)	139 (98.6%)	199 (99.0%)
Grade 1	0 (0%)	2 (1.4%)	2 (1.0%)
Body temperature in C degree (**)			
Mean (SD)	36.5 (0.4)	36.7 (0.4)	36.7 (0.4)
Median (min–max)	36.6 (36–37)	36.8 (36–38)	36.8 (36–38)
**Drowsiness**	0 (0%)	0 (0%)	0 (0%)
**Headache**	0 (0%)	0 (0%)	0 (0%)
**Irritation**	0 (0%)	0 (0%)	0 (0%)
**Fatigue**	0 (0%)	0 (0%)	0 (0%)

(*): *p* < 0.05; (**): *p* < 0.01; Grade 0 = absence; redness and swelling diameter were measured if present; body temperature was measured for all participants.

**Table 3:** Temperature and pulse of the participants at screening time (D0) and the end of the study (D42).

	At Screening Time (D0)			At the End of the Study (D42)			*p*-Value for D0–D42 Comparison		
	1–5 yrs (n = 60)	6–12 yrs (n = 141)	Total (n = 201)	1–5 yrs (n = 60)	6–12 yrs (n = 141)	Total (n = 201)	1–5 yrs	6–12 yrs	Total
**Temperature, °C**									
Mean (SD)	36.4 (0.2)	36.4 (0.2)	36.4 (0.2)	36.8 (0.2)	36.8 (0.2)	36.8 (0.2)	<0.0001	<0.0001	<0.0001
Median (min–max)	36.4 (36–37)	36.4 (36–37)	36.4 (36–37)	36.8 (36–37)	36.8 (36–37)	36.8 (36–37)			
**Pulse (beats/min)**									
Mean (SD)	94.1 (17.0)	85.5 (17.3)	88.1 (17.6)	100.8 (9.1)	91.9 (8.0)	94.6 (9.3)	0.008	0.0001	<0.0001
Median (min–max)	97.0 (56–136)	84.0 (51–129)	87.0 (51–136)	99.0 (85–127)	90.0 (80–129)	95.0 (80–129)			

**Table 4:** Distribution of solicited adverse events within 7 days following vaccination.

Adverse Events	1–5 Years Old (n = 60)	6–12 Years Old (n = 141)	Total (n = 201)
**Pain at injection site**	6 (10.0%)	30 (21.3%)	36 (17.9%)
Grade 0	54 (90.0%)	111 (78.7%)	165 (82.1%)
Grade 1	6 (10.0%)	30 (21.3%)	36 (17.9%)
**Redness**	0 (0%)	8 (5.7%)	8 (4.0%)
Grade 0	60 (100%)	135 (95.7%)	195 (97.0%)
Grade 1	0 (0%)	4 (2.8%)	4 (2.0%)
Grade 2	0 (0%)	2 (1.4%)	2 (1.0%)
Grade 3	0 (0%)	0 (0%)	0 (0%)
**Swelling**	4 (6.7%)	21 (14.9%)	25 (12.4%)
Grade 0	58 (96.7%)	124 (87.9%)	182 (90.5%)
Grade 1	2 (3.3%)	16 (11.3%)	18 (9.0%)
Grade 2	0 (0%)	1 (0.7%)	1 (0.5%)
Grade 3	0 (0%)	0 (0%)	0 (0%)
**Fever**	1 (1.7%)	2 (1.4%)	3 (1.5%)
Grade 0	59 (98.3%)	139 (98.6%)	198 (98.5%)
Grade 1	1 (1.7%)	2 (1.4%)	3 (1.5%)
**Drowsiness**	0 (0%)	0 (0%)	0 (0%)
**Headache**	0 (0%)	1 (0.7%)	1 (0.5%)
**Irritation**	0 (0%)	0 (0%)	0 (0%)
**Fatigue**	0 (0%)	1 (0.7%)	1 (0.5%)
**Varicella-like Rash**	1 (1.7%)	0 (0%)	1 (0.5%)
**Varicella-like Rash Severity**			
Mild	1 (1.7%)	0 (0%)	1 (0.5%)

Grade 0 = Absence.

**Table 5:** Rate and severity of unsolicited adverse events by age group.

**Unsolicited Adverse Events**	**1–5 Years Old** **(n = 60)**	**6–12 Years Old** **(n = 141)**	**Total** **(n = 201)**
**Any adverse events**	**6 (10%)**	**2 (1.42%)**	**8 (3.98%)**
Mild	4 (6.66%)	0 (0.00%)	4 (1.99%)
Moderate	2 (3.33%)	2 (1.42%)	4 (1.99%)

**Table 6:** Seroconversion rates by age group (n = 35).

	1–5 Years Old (n = 10)	6–12 Years Old (n = 25)	Total (n = 35)
Seroconversion ratio	10/10	25/25	35/35
Seroconversion rate	100%	100%	100%

**Table 7:** Geometric mean titer (GMT) and CI 95% with FAMA Test.

Time Point	D0	D42	*p*-Value
Mean (SD)	1.37 (1.97)	18.02 (2.22)	*p* < 0.001
95% CI	0.83–2.26	10.28–31.60	

FAMA, fluorescent antibody-to-membrane antigen.

## Data Availability

Data is available at corresponding authors upon reasonable request.
